# SuperSelective PCR primers for determining *cis/trans* configurations of mutations within the same gene

**DOI:** 10.1371/journal.pone.0349389

**Published:** 2026-05-18

**Authors:** Diana Yaneth Vargas, Fred Russell Kramer

**Affiliations:** Public Health Research Institute, New Jersey Medical School, Rutgers University, Newark, New Jersey, United States of America; Nelson Mandela African Institute of Science and Technology, UNITED REPUBLIC OF TANZANIA

## Abstract

In non-small cell lung cancer, the choice of an effective targeted therapy often depends on the presence of a somatic mutation that alters an amino acid in a key protein. However, if an additional somatic mutation occurs in the same mutant gene, causing an additional amino acid substitution to occur in that protein, the targeted therapy can become ineffective. A PCR assay has now been developed that utilizes two different SuperSelective primers, one primer for the amplification of the DNA strand containing the original somatic mutation, and a second primer for the amplification of the DNA strand containing the additional somatic mutation. Exponential amplification only occurs if these two somatic mutations occur in *cis* on the DNA molecule present in the same sister chromosome, and does not occur at all if the two different somatic mutations occur in *trans* on different DNA molecules in two different sister chromosomes. Significantly, if only one of these two mutations is present, amplification does not occur, thereby ensuring that the assay specifically detects the coexistence of both target mutations being present on the same DNA strand. This assay (demonstrated utilizing *EGFR* exon 20 mutations T790M and C797S) is extraordinarily sensitive, enabling the early substitution of a more effective therapy if the two mutations occur in a *cis* configuration. Moreover, these SuperSelective PCR assays can utilize DNA fragments isolated from noninvasive liquid biopsy samples, and they can be carried out on widely available spectrofluorometric thermal cyclers.

## Introduction

In non-small cell lung cancer (NSCLC), the choice of an effective targeted therapy can depend on the presence of a sensitizing mutation in a gene that alters an amino acid in the encoded protein. However, if an additional mutation subsequently arises in the same mutant gene that causes an additional amino acid substitution to occur in that protein, the original therapy may become ineffective. Examples of this situation occur in the epidermal growth factor receptor (*EGFR*) gene [1], in the anaplastic lymphoma kinase (*ALK*) gene [2], and in the Kirsten rat sarcoma viral oncogene homolog (*KRAS*) gene [3].

Although next-generation sequence analysis and droplet digital PCR can be utilized to detect the early occurrence of such mutations [4], those techniques require specialized equipment and expertise, and their sensitivity is compromised by the generation of false-positive signals from the simultaneous amplification of the abundant wild-type DNA present in the sample being analyzed. It is therefore desirable to develop assays that can be carried out on widely available PCR instruments that do not amplify the wild-type DNA fragments that are present in the DNA sample being analyzed. Moreover, current real-time PCR assays that utilize DNA fragments isolated from a patient’s blood plasma sample cannot determine whether additional somatic mutations occur in *cis* on the same sister chromosome as the original somatic mutation (which would compromise the previous targeted therapy), or whether the additional somatic mutation occurs in *trans* on a different sister chromosome (in which case the previous targeted therapy would still be effective).

For example, *EGFR* mutation L858R in lung cells causes the *EGFR* protein to constitutively activate its tyrosine kinase activity, which leads to the uncontrolled phosphorylation of effector proteins, resulting in the proliferation of those cells, causing cancer [5]. Treatment with gefitinib inhibits the tyrosine kinase activity of the mutant *EGFR* protein, preventing proliferation of the mutated cancer cells [6]. When an additional *EGFR* mutation occurs in response to gefitinib treatment (such as T790M in exon 20) altering the shape of the ATP-binding site in the *EGFR* protein, then the use of osimertinib is needed to control the proliferation of those cells [7]. However, if in addition to the occurrence of the T790M mutation and after subsequent treatment with osimertinib, a C797S mutation also occurs in exon 20 on the same sister chromosome containing the T790M mutation, (i.e., in *cis*), then osimertinib is ineffective [8]. On the other hand, if the T790M and C797S mutations occur in *trans* on different sister chromosomes, osimertinib may still maintain at least partial activity [9]. Currently, a number of different drugs are being explored for treating patients with the T790M and C797S mutations present in *cis* on the same sister chromosome, but none have been completely effective [10–12].

Here we describe a general strategy to distinguish whether two different mutations occur in *cis* or *trans* utilizing two SuperSelective PCR primers [13–16], each of which is designed to initiate amplification only on DNA fragments containing a targeted DNA mutation, while preventing amplification on DNA fragments containing the related wild-type DNA sequence. In this assay, one SuperSelective primer is designed to amplify the DNA strand containing the original somatic mutation, and the other SuperSelective primer is designed to amplify the complementary DNA strand containing the new somatic mutation. The result of using this pair of primers is that exponential amplification only occurs if the two different somatic mutations occur in *cis*, and does not occur if the two different somatic mutations occur in *trans*. The resulting assay is very sensitive, potentially enabling the early detection of resistance mutations and intervention with an effective targeted therapy.

SuperSelective PCR primers are extraordinarily specific because they possess three different functional segments: (i) an “anchor sequence” at their 5’ end, whose length is similar to the length of a conventional PCR primer, thereby assuring that the SuperSelective primer binds exclusively to DNA fragments containing the gene of interest, irrespective of whether that gene fragment possesses or does not possess the mutation of interest; (ii) a short “foot sequence” at their 3’ end that will only anneal under assay conditions to the complementary mutant target sequence if it is present in the target gene to which the SuperSelective primer is bound, thereby initiating amplification, but will not anneal to the mismatched closely related wild-type target sequence if it is present in the target gene to which the SuperSelective primer is bound, thereby preventing amplification; and (iii) a single-stranded “bridge sequence” that separates the 5’-anchor sequence from the 3’-foot sequence, and which is chosen by the designer of the SuperSelective primer to be located across from an “intervening sequence” in the target gene that occurs between the target of the anchor sequence and the potential target of the foot sequence, and is designed to form a single-stranded “bubble” that separates the gene-detection function of the anchor sequence from the mutation-detection function of the foot sequence.

**Fig 1:**
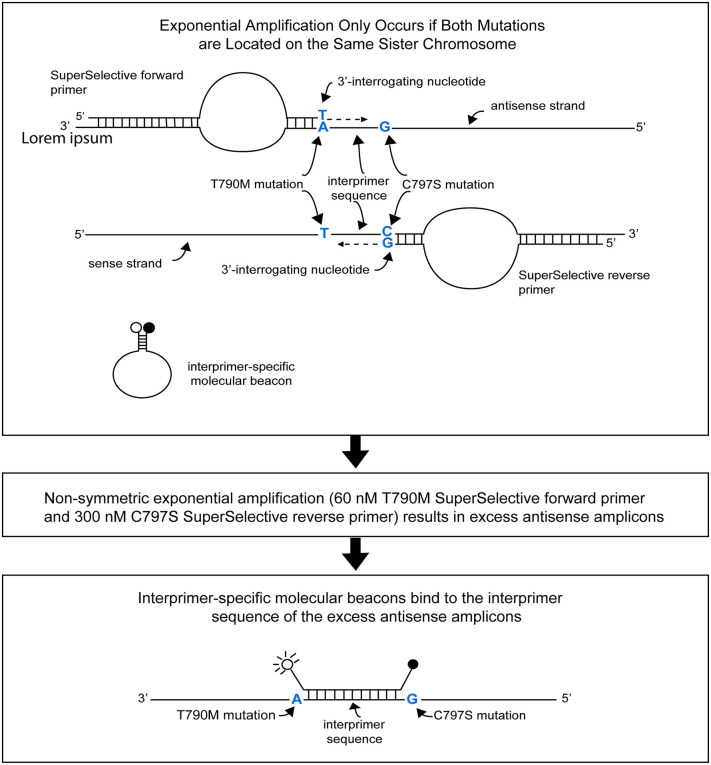
Design of a PCR assay that utilizes a pair of SuperSelective primers for the specific detection of two different *EGFR* mutations (T790M and C797S) located 20 nucleotides apart (in *cis*) on the DNA molecule present in the same sister chromosome.

Our design of a PCR assay that utilizes a pair of SuperSelective primers to distinguish whether two different *EGFR* exon 20 mutations (T790M and C797S) are located on separate sister chromosomes (in *trans*) or are located 20 nucleotides apart from each other on the same sister chromosome (in *cis*) is shown in Fig 1. A key aspect of this assay is that the SuperSelective primers do not amplify wild-type DNA fragments that lack both mutations (i.e., wild-type target strands). And most importantly, if a DNA target fragment possesses only one of the two mutations, although a SuperSelective primer will initiate synthesis on that DNA fragment, the resulting amplicon will not possess a copy of the other mutation, so the other SuperSelective primer will not be able to initiate synthesis on that amplicon. Consequently, exponential amplification only occurs in these assays on DNA fragments that possess both mutations in a *cis* configuration.

Another significant aspect of these SuperSelective PCR assays is that once synthesis is initiated, and a copy of the template strand is produced, and once that copy serves as a template for the synthesis of its complementary amplicon, each of these amplicons consequently possess complete copies of the entire SuperSelective primer sequence that initiated their amplification. So after the initial recognition events, the entire sequence of each SuperSelective primer binds to its amplicon, and exponential amplification is efficient and as highly productive as a PCR assay that utilizes conventional primers.

In the assays described in this report, the SuperSelective *EGFR* T790M forward primers bind to the *EGFR* antisense DNA strands; and if (and only if) those strands contain the T790M mutation, then those primers are extended by the DNA polymerase to generate amplicons that may (or may not) contain the C797S mutation. Similarly, the SuperSelective *EGFR* C797S reverse primers bind to the *EGFR* sense DNA strands; and if (and only if) those strands contain the C797S mutation, then those primers are extended by the DNA polymerase to generate amplicons that may (or may not) contain the T790M mutation. If both the T790M mutation and the C797S mutation are present on the same sister chromosome (i.e., they occur in *cis*), then exponential amplification occurs. On the other hand, if the T790M mutation and the C797S mutation are present on different sister chromosomes (i.e., they occur in *trans*), then exponential amplification does not occur, and the signal from the molecular beacon probes present in the assay is essentially dark.

Furthermore, in these assays, by design, the initial concentration of the SuperSelective forward primer is significantly lower than the initial concentration of the SuperSelective reverse primer. Consequently, as exponential amplification proceeds, the limited concentration of the Super Selective forward primer is used up, and the excess concentration of the SuperSelective reverse primer continues to catalyze the linear synthesis of excess antisense amplicons, thereby enabling interprimer-specific molecular beacon probes to bind to those excess antisense amplicons, generating a fluorescence signal, with little competition from the limited number of sense amplicons [17].

These *cis*-mutation detection assays are particularly sensitive, because by design they are highly unlikely to generate false-positive *cis* amplicons. There are at least three reasons for this attribute: (1) SuperSelective primers have been shown to selectively amplify a rare number of mutant templates in the presence of a large number of closely related wild-type templates [13–16]; (2) the probability that a DNA polymerase will mis-incorporate a false mutant target nucleotide at a particular position during PCR is approximately only 1 in 100,000 nucleotide additions [18]; and (3) liquid biopsy samples analyzed by PCR usually contain fragments from only a few thousand human genomes, and only a small fraction of those genomes originate from tumor cells [3].

## Materials and methods

### SuperSelective PCR Primers and the Interprimer-specific Molecular Beacon Probe

Table 1 shows the sequences of SuperSelective forward primer 24–22/13–8:1, SuperSelective reverse primer 30–20/19–8:1, and the interprimer-specific molecular beacon probe that was used in these assays for the detection of *EGFR* exon 20 mutations T790M and C797S. The SuperSelective primers were purchased from Integrated DNA Technologies (Coralville, IA), and the interprimer-specific molecular beacon probe was purchased from LGC Biosearch Technologies (Petaluma, CA). The concentration of the solutions containing each primer, and the concentration of the solution containing the molecular beacon, was determined in a NanoDrop spectrophotometer (Thermo Fisher Scientific, Waltham, MA).

**Table 1:** Nucleotide Sequences of the SuperSelective Primers and the Molecular Beacon Probe.

***EGFR* exon 20 T790M SuperSelective Forward Primer 24–22/13–8:1**
5´-GCCGCCTGCTGGGCATCTGCCTCA-AAGAATCAACAAGCTACAACTC-**GCTCATCAT**-3´
***EGFR* exon 20 C797S SuperSelective Reverse Primer 30–20/19–8:1**
5´-TTGAGCAGGTACTGGGAGCCAATATTGTCT-GTCCTTTACAAGCACGAGTG-**CCAGGAGGG**-3´
**Interprimer-specific Molecular Beacon Probe**
5’-(Fluorescein)-CCGTCG-*CAGCTCATGCCCTTCGGC*-CGACGG-(Black Hole Quencher 1)-3’

In each SuperSelective primer, the underlined nucleotides indicate the bridge sequence chosen by the primer designer, and the bold nucleotides indicate the foot sequence that includes a 3’-interrogating nucleotide that must be complementary to the mutation in the target sequence for amplification to occur during the initial annealing cycles. The italicized nucleotides in the molecular beacon are complementary to the interprimer sequence in the amplicon to which it binds.

### DNA target fragments generated from plasmids

Four different target plasmids, each consisting of a pIDTSmart Amp vector containing a synthetic 200-bp gene fragment encompassing a different *EGFR* target sequence, were purchased from Integrated DNA Technologies. The target sequences were: (i) T790M only, (ii) C797S only, (iii) T790M and C797S in *cis*; and (iv) wild-type containing neither T790M nor C797S. Each plasmid was approximately 3,000 bp long and contained a single endonuclease ScaI restriction site located away from the inserted sequence. Approximately 4 μg of each plasmid was separately digested for 120 minutes at 37°C in 20 μL containing 10 units of ScaI (New England Biolabs, Ipswich, MA) in a buffer provided by New England Biolabs that contained 10 mmol/L MgCl_2_, 100 mmol/L NaCl, 1 mmol/L dithiothreitol, and 50 mmol/L Tris-HCl (pH 7.9); followed by incubation for 20 minutes at 80°C to inactivate the enzyme. The concentration of each linearized plasmid was determined in a NanoDrop spectrophotometer, after which the plasmids were substantially diluted with water to generate stock solutions of target fragments for use in the assays. Furthermore, the final concentration of each plasmid preparation was quantified by droplet digital PCR utilizing T790M or C797S controls purchased from Bio-Rad Laboratories (Hercules, CA).

### Human genomic wild-type DNA target fragments

Wild-type human genomic DNA (from multiple anonymous donors), catalog number G1521, was purchased from the Promega Corp. (Madison, WI). Approximately 9 μg of this DNA was digested for 120 minutes at 37°C in 50 μL containing 10 units of restriction endonuclease DraI (New England Biolabs) in a buffer provided by New England Biolabs that contained 100 μg/mL bovine serum albumin, 10 mmol/L magnesium acetate, 50 mmol/L potassium acetate, and 20 mmol/L Tris-acetate (pH 7.9); followed by incubation for 20 minutes at 65°C to inactivate the enzyme. The resulting mixture of genomic DNA restriction fragments was analyzed by digital PCR to determine the concentration of *EGFR* wild-type DNA target fragments, after which that mixture was diluted with water to generate a stock solution of wild-type *EGFR* target fragments for use in the assays. The lengths of these *EGFR*-containing DraI fragments, all of which contained intact *EGFR* target sequences, varied from 1,000–3,000 bp.

### Polymerase chain reaction assay conditions

All of the PCR amplifications were performed in 30 μL volumes that contained: 50 mmol/L KCl, 2.5 mmol/L MgCl_2_, 10 mmol/L Tris-HCl (pH 8.0), 250 μmol/L deoxy-ATP, 250 μmol/L deoxy-CTP, 250 μmol/L deoxy-GTP, 250 μmol/L deoxy-TTP, 0.5% of the nonionic surfactant Tween 20 (Sigma Aldrich, St. Louis, MO), 50 mmol/L tetramethylammonium chloride (Sigma Aldrich), and 1.5 units Platinum *Taq* DNA polymerase (Thermo Fisher Scientific). These amplifications were carried out in 0.2 mL white polypropylene reaction tubes (USA Scientific, Ocala, FL) in a Bio-Rad Laboratories CFX-96 Touch spectrofluorometric thermal cycler. The thermal cycling program was 2 minutes at 95°C, followed by 55 cycles of 95°C for 20 sec, 60°C for 20 sec, and 72°C for 20 sec. Molecular beacon fluorescence intensity was automatically measured by the thermal cycler at the end of each 60°C annealing cycle.

### Assays that demonstrate *cis-trans* specificity

Four replicate real-time PCR amplifications of each of three different digested plasmid target mixtures were carried out simultaneously. Each reaction contained 60 nmol/L *EGFR* T790M SuperSelective forward primer, 300 nmol/L *EGFR* C797S SuperSelective reverse primer, and 300 nmol/L of the fluorescein-labeled interprimer-specific molecular beacon probe. All reactions contained 10,000 copies of the *EGFR* wild-type target templates. One set of four reactions additionally contained 10 copies of the *EGFR* T790M-C797S (*cis*) target templates in each reaction; a second set of four reactions additionally contained 10 copies of the *EGFR* T790M target templates and 10 copies of the *EGFR* C797S target templates (*trans*) in each reaction; and a third set of four reactions contained no copies of either mutant target template; that is, only wild-type templates were present in the third set of four reactions.

### Assays that demonstrate *cis-trans* sensitivity

Eight replicate real-time PCR amplifications of each of four groups of samples (i.e., 32 reactions were carried out simultaneously), where each sample contained 20,000 human genomic wild-type DNA fragments, and the individual samples in each of four groups contained either 1,000, 100, 10, or 0 copies of the *EGFR* T790M-C797S (*cis*) target template. In addition, as a wild-type control [16], each sample contained 60 nmol/L of a SuperSelective forward PCR primer and 500 nmol/L of a conventional reverse PCR primer, as well as 50 nmol/L of a Quasar 705-labeled interprimer-specific molecular beacon probe, for the amplification and detection of the *β-actin* reference gene present in wild-type human genomic DNA.

## Results

### Design of the *cis-trans* Assay

**Fig 2:**
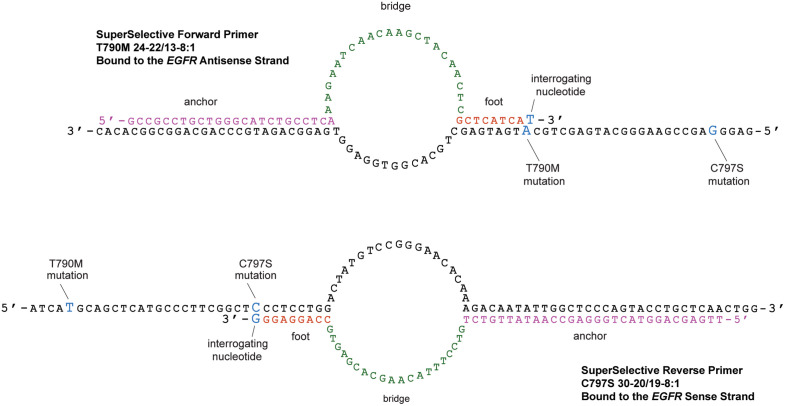
Hybridization of a pair of SuperSelective primers to *EGFR* exon 20 mutations T790M and C797S present in *cis* within both strands of the DNA molecule present in the same sister chromosome.

Fig 2 shows the nucleotide sequences of the two SuperSelective primers bound to their mutant target DNA sequences in these assays. SuperSelective forward primer *EGFR* T790M 24–22/13–8:1 possesses a 24 nucleotide-long anchor sequence, a 22 nucleotide-long bridge sequence (which forms a single-stranded bubble with a 13 nucleotide-long intervening sequence), and a 9 nucleotide-long foot sequence with a 3’-interrogating nucleotide. Similarly, the design of SuperSelective reverse primer C797S is summarized as 30–20/19–8:1. *EGFR* T790M mutant DNA possesses an A:T base pair in place of the wild-type G:C base pair (thereby encoding a methionine in place of a threonine); and *EGFR* C797S mutant DNA possesses a G:C base pair in place of the wild-type C:G base pair (thereby encoding a serine in place of a cysteine).

### High Specificity of the *cis-trans* Assay

The underlying aspect of utilizing a pair of SuperSelective primers in PCR assays is that both of those primers should completely ignore the presence of closely related abundant wild-type DNA sequences, so that amplification only occurs if the mutant target DNA sequences are present in *cis* within the same gene. To explore this potential, we first performed preliminary PCR assays containing both *EGFR* T790M SuperSelective forward primers and *EGFR* C797S SuperSelective reverse primers with either: (a) 10,000 copies of a linearized plasmid containing only the T790M mutation; or (b) 10,000 copies of a linearized plasmid containing only the C797S mutation; or (c) 10,000 copies of a linearized plasmid containing only the T790M mutation as well as 10,000 copies of a linearized plasmid containing only the C797S mutation; or (d) 10,000 copies of a linearized plasmid containing both of these mutations in *cis*. The first three reactions yielded no signal, whereas the last reaction containing both mutations in *cis* yielded a robust fluorescent signal.

Encouraged by this result, we carried out a series of 12 PCR assays in which each sample that was tested also contained 10,000 wild-type DNA target fragments. Four of the samples also contained 10 DNA target fragments that possessed both the *EGFR* T790M and C797S mutations within the same sequence (i.e., in a *cis* configuration). Four other samples contained 10 DNA target fragments that only possessed the T790M mutations and 10 more DNA target fragments that only possessed the C797S mutation (i.e., this target mixture mimicked a *trans* configuration). And the last four samples only contained the 10,000 wild-type DNA fragments.

**Fig 3:**
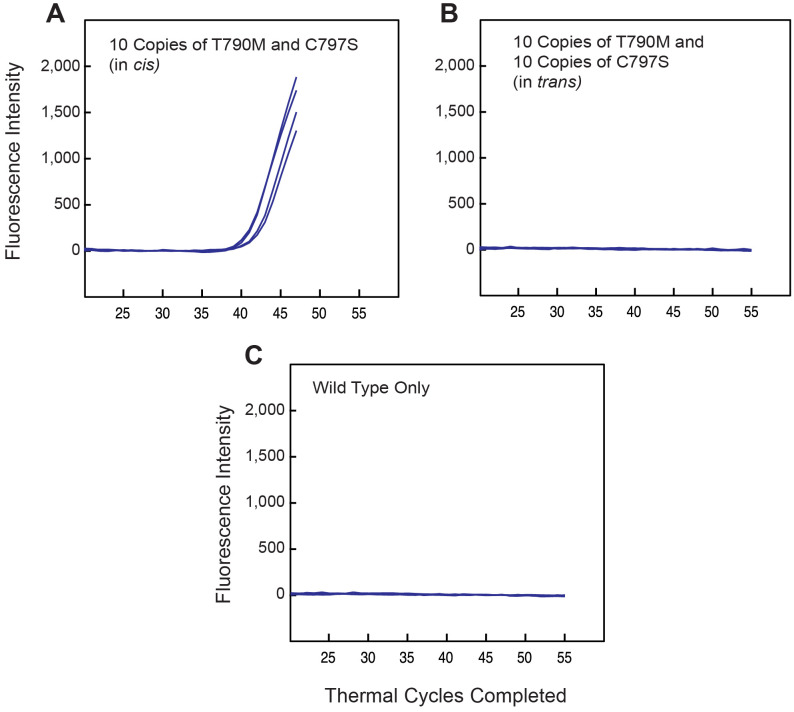
Demonstration of the selectivity of a *cis*-specific pair of SuperSelective primers for the amplification of *EGFR* mutations T790M and C797S in assays containing 10,000 copies of the *EGFR* wild-type sequence. Only target DNA sequences containing these mutations in a *cis* configuration gave rise to detectable amplicons. **A.** Mutations present in *cis*; **B.** mutations present in *trans*; and **C.** mutations not present.

Fig 3 shows the results of the real-time PCR assays carried out with these 12 samples. Only the samples containing both mutations in a *cis* configuration gave rise to a fluorescent signal from the molecular beacon probes. No signal arose when the two mutations were in a *trans* configuration. And most importantly, no signal arose from the samples that each contained only 10,000 closely related wild-type DNA fragments. These results confirm the extraordinary specificity of SuperSelective primers.

### Sensitivity of the *cis-trans* Assay in the Presence of Human Genomic DNA

An additional aspect of utilizing these SuperSelective primer pairs in real-time PCR assays is that they provide results that enable a determination of the relative abundance of the DNA target fragments in a sample, even if the sample contains DNA fragments from the entire human genome. In order to demonstrate this ability, a series of 32 samples were prepared in which each sample that was tested contained DNA fragments prepared from 20,000 wild-type human genomes. In addition, eight of the samples contained 1,000 *EGFR* T790M-C797S *cis* DNA mutant fragments; eight contained 100 *cis* DNA mutant fragments; eight contained only 10 *cis* DNA fragments; and eight did not contain any *cis* DNA fragments. All of the assays also contained a second set of primers and a differently colored molecular beacon probe for the detection of the wild-type *β-actin* reference gene in order to confirm the simultaneous presence in each assay of the DNA fragments from 20,000 human genomes [16].

**Fig 4:**
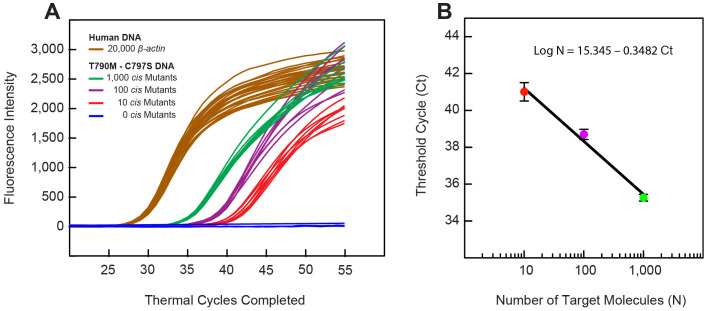
Demonstration of the sensitivity of real-time PCR assays that utilize a pair of SuperSelective primers for the detection of *EGFR* mutations T790M and C797S located in *cis.* Four groups of eight reactions were carried out simultaneously, each of which contained 20,000 copies of the entire wild-type human genome (whose *β-actin* sequence served as a reference gene), and each of the four groups contained either 1,000, 100, 10, or 0 copies of a linearized plasmid possessing the target mutations in *cis*. **A.** Samples that only possessed human wild-type sequences did not produce an *EGFR* signal. **B.** Graph of the mean threshold cycle values (Ct) of the reactions containing T790M-C797S*cis* target DNAs plotted against the logarithm of the number of mutant targets in each group resulted in a least-square fitted straight line (with an R^2^ coefficient of determination of.987), whose equation accurately reflects the number of targets present in each reaction. Bars above and below each point reflect the standard deviation of each mean Ct value.

Fig 4 shows the results of the real-time PCR assays carried out with these 32 samples. In these assays, exponential amplification occurred until the limited T790M primers were used up, after which the excess C797S primers continued linear amplification. The point at which this transition occurred (the “threshold cycle,” abbreviated “Ct”) was automatically calculated from the fluorescence intensity data at each thermal cycle by the Bio-Rad CFX Maestro computer program. The eight samples that contained 1,000 T790M-C797S *cis* mutations each, gave an average Ct value of 35.27 with a standard deviation of +/- 0.183 Ct; the eight samples that contained 100 T790M-C797S *cis* mutations each, gave an average Ct value of 38.70 with a standard deviation of +/- 0.276 Ct; and the eight samples that contained only 10 T790M-C797S *cis* mutations each, gave an average Ct value of 41.01 with a standard deviation of +/- 0.498 Ct. The more *EGFR* T790M-C797S *cis* DNA mutant fragments that were originally present in a sample, the earlier the threshold cycle that occurred. These results confirm that the threshold cycle reflects the number of target DNA fragments that were originally present, even if there is only a very low number of *cis* mutations in the sample. Furthermore, these results demonstrate a nominal limit of detection (LOD) of ten copies of *EGFR* T790M-C797S *cis* DNA mutant fragments.

Significantly, samples that did not contain any T790M-C797S *cis* DNA fragments did not produce a signal; yet samples that contained only 10 T790M-C797S *cis* DNA fragments gave a positive signal, confirming the excellent sensitivity of these *cis-trans* assays. In these assays, the logarithm of the number of target DNA molecules originally present in each sample is inversely proportional to the observed threshold cycle [19,20], confirming that assays employing pairs of SuperSelective PCR primers can be used routinely to determine the presence and abundance of *EGFR* T790M-C797S *cis* DNA mutant fragments.

Furthermore, these results confirm that when using these SuperSelective primer pairs no false-positive signals arise from the very abundant human genomic wild-type DNA fragments. Yet, positive signals were generated from very rare T790M-C797S *cis* DNA fragments even in the presence of these very abundant wild-type fragments. The key implication of these observations is that these SuperSelective primer pairs can be used with DNA fragments obtained from human liquid biopsy samples to detect and quantify T790M-C797S *cis* DNA fragments.

## Discussion

This approach is not limited to the detection and quantitation of the *EGFR* T790M and C797S mutations that were used in these demonstration assays. It can be utilized for the detection of a wide range of other clinically relevant *cis*-*trans* mutation configurations that arise in precision oncology. For example, NSCLC patients who possess the *ALK* G1202R mutation often respond to treatment with lorlatinib, but this drug fails to work if an additional *ALK* mutation occurs in *cis*, such as S1206Y (9 nucleotides away), or L1196M (18 nucleotides away), or F1174C (82 nucleotides away) [2]. All four of these mutations occur in *ALK* exon 23, which encodes a portion of the kinase domain of the encoded *ALK* protein. Based on the design of the *EGFR* T790M-C797S *cis* assay, three corresponding SuperSelective PCR primer pair assays could be implemented to detect the *cis ALK* mutation pairs *ALK* G1202R-S1206Y, *ALK* G1202R-L1196M, and *ALK* G1202R-F1174C. A positive result would indicate the presence of two mutations in *cis*, suggesting the need for an alteration in therapy. Moreover, there are other similar *cis* pairs that occur in other types of cancer whose sensitive detection could potentially improve the course of therapy.

Significantly, these results imply that PCR assays that utilize SuperSelective primer pairs can be used to identify patients possessing a small amount of a relevant *cis* mutation pair, and those patients will be ideal for assessing the effectiveness of potential new targeted therapies, resulting in the identification of a therapy that works well. Moreover, these assays can then be used frequently for early detection in cancer patients possessing *cis* mutations that might require a new targeted therapy. And finally, these assays should be able to be used frequently over time, utilizing non-invasive liquid biopsies, to assess the effectiveness of the therapy, hopefully extending the life of the patient.

The *EGFR* T790M-C797S example described here involves two single-nucleotide polymorphisms. However, SuperSelective PCR primers can also be utilized to detect a broad range of other genomic alterations, including clinically relevant insertions, deletions, and gene fusions [21]. The key characteristic of SuperSelective primer designs is their exceptional specificity enabled by combining the gene recognition function of their anchor sequence with the independent mutation recognition function of their short foot sequence. Consequently, by design, amplification is only initiated if the primer’s target mutation is present within the DNA to which it is bound. Thus, the use of pairs of SuperSelective primers that are highly unlikely to initiate synthesis on closely related wild-type templates, potentially enable the construction of a wide variety of highly specific and extremely sensitive assays that reliably detect relevant mutant alleles.

Furthermore, because the identification of the resulting amplicons in these assays is not dependent on the discriminatory ability of probes, but rather depends on the enhanced discriminatory ability of the SuperSelective primers, these assays should improve the sensitivity of digital PCR as well as improving the sensitivity of real-time PCR.

In summary, the use of pairs of SuperSelective PCR primers (one primer specific for a particular somatic mutation and the other primer specific for a different somatic mutation present in *cis* within the same gene), provides a sensitive PCR assay method, because by design closely related wild-type templates are not exponentially amplified. Even if a rare mispriming event occurs on a wild-type template, the resulting amplicon will not serve as a template for exponential synthesis because it will only possess one of the two mutations required for exponential synthesis to occur.

The assays described here are demonstration assays that utilized endonuclease-digested wild-type human genomic DNA and linearized plasmid-derived mutant templates combined into samples with well-defined allelic frequencies that served as surrogates for circulating cell-free human DNA samples. These controlled samples enabled us to rigorously assess the performance, selectivity, sensitivity, and reproducibility of SuperSelective PCR primer-pair assays. In the future, clinical diagnostic laboratories that have access to well characterized patient-derived cell-free human DNA samples will be able to determine the practical effects of any biological heterogeneity and pre-analytical variability on the translational utility of this method.

In conclusion, SuperSelective PCR primers provide a general means for the identification of whether pairs of mutations occur in *cis* or *trans*. Early detection of *cis* resistance mutations can potentially guide treatment choices, improve disease monitoring through the use of noninvasive liquid biopsies, and is likely to improve effective personalized cancer therapy.
